# Is Population Density a Risk Factor for Communicable Diseases Like COVID-19? A Case of Bangladesh

**DOI:** 10.1177/1010539521998858

**Published:** 2021-03-06

**Authors:** Md. Zakiul Alam

**Affiliations:** 1 University of Dhaka, Dhaka, Bangladesh

**Keywords:** Bangladesh, COVID-19, population density, population density and COVID-19, urbanization and COVID-19

## Abstract

Bangladesh is one of the most densely populated countries in the world struggling to prevent COVID-19 (coronavirus disease 2019). This study employed correlation, cluster analysis, and multiple linear regression analyses using district-wise COVID-19 infection and socioeconomic data. It is observed that there is a strong positive correlation (*r* = 0.876, *P* < .001) between population density and COVID-19, explaining a 60% variation in Bangladesh. The relationship between urbanization and COVID-19 is also positively strong (*r* = 0.802, *P* < .001). Urban settlements have a higher risk of spreading diseases due to the enormous population density. For future planning to prevent COVID-19 and other related infectious diseases, population density should be considered a risk factor.

## What We Already Know

• Rising population density increases the COVID-19 infection in Algeria.

• Connectivity and metropolitan size matter more than density in the spread of the COVID-19 pandemic in the USA.

## What This Article Adds

• There is a strong positive correlation between population density and COVID-19 infection in Bangladesh.

• The urban settlements have a higher risk of spreading diseases due to the enormous population density and close connectivity in Bangladesh.

• For future planning to prevent the spread of COVID-19 and other related infectious diseases like Dengue in Bangladesh, population density should be considered a risk factor.

## Introduction

The World Health Organization declared the severe acute respiratory syndrome coronavirus 2 (SARS-CoV-2), the coronavirus disease 2019 (COVID-19), as a global pandemic on March 11, 2020. There have been 31.7 million confirmed cases of COVID-19 and 972 000 deaths globally as of September 23, 2020. Bangladesh has struggled with this disease for the last few months. Since COVID-19 is an infectious disease, living in close contact may increase the spread. Previous studies have shown that rising the population density increases the COVID-19 infection.^1^ Hamidi and colleagues^2^ found that connectivity matters more than density in the spread of the COVID-19 pandemic. In contrast, metropolitan size matters more than population density in the spread of the pandemic in the United States.^3^

Bangladesh is one of the most densely populated countries in the world. As a result, the preparedness responses to COVID-19 may need to consider some unique dynamics of Bangladesh. To reduce the numbers of COVID-19 confirmed cases and flatten the curve of the infection in the absence of a vaccine and explicit treatment, Bangladesh has taken nontherapeutic measures, including full (or partial lockdown), risk zone-based lockdowns, maintaining social isolation, limiting working hours, closing all institutions, restaurants and, shopping malls, and restricting the movement of people through travel bans.^4-6^ However, these preventive measures may not have been as effective as they have been in other countries due to higher population density and diverse socioeconomic conditions. In urban areas, where there are a higher population density and connectivity, there is a great risk of spreading the virus. This article aims to explore the association between population density and the incidence of COVID-19 infection in Bangladesh.

## Methods

There are 64 districts in Bangladesh. District-wise COVID-19 infection data were extracted from the Directorate General of Health Services website of Bangladesh on September 18, 2020.^7^ The population and socioeconomic data including population density, urbanization rate, activity rate, and literacy rate were taken from population and housing census of Bangladesh.^8^ Poverty rate was taken from household income and expenditure survey 2016. Cluster analysis (hierarchical) was used to classify districts by the number of COVID-19 confirmed cases and population density. Pearson correlation coefficients were measured at the bivariate level. To control the effects of other socioeconomic variables on COVID-19, we utilized multiple linear regression.

## Results

**Figure 1.**
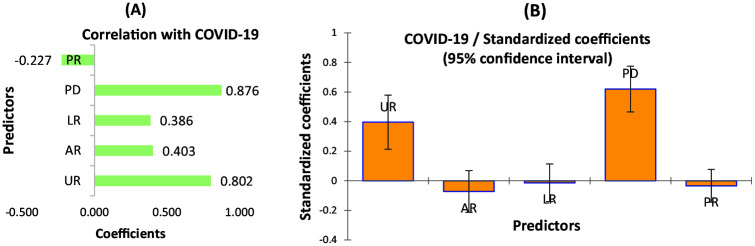
(A) The Pearson correlation between confirmed COVID-19 cases and selected socioeconomic predictors. (B) Standardized coefficients for COVID-19 by selected socioeconomic variables in Bangladesh (n = 64 districts of Bangladesh). PD, population density; UR, urbanization rate; AR, activity rate; LR, literacy rate; PR, poverty rate.

The population density of Bangladesh is 1125 population per square kilometer (km). The mean number of COVID-19 confirmed cases were 3992 in 64 districts. Cluster analysis divided the districts into 4 groups: group 1 (35 districts), group 2 (17 districts), group 3 (10 groups including major cities), and group 4 (2 districts including Dhaka and Chattogram). The average number of cases were 1070, 2784, 5988, and 55 398 for groups 1, 2, 3, and 4, respectively. There was a strong positive correlation (*r* = 0.876, *P* < .001) between the population density and confirmed cases in Bangladesh (Figure 1A). However, the cluster-specific analysis showed that there was a relatively weak correlation for groups 1 and 2. For group 3, it was moderate, while for group 4, it was a very strong correlation.

Like population density, the relationship between urbanization and COVID-19 infection cases were also strong (*r* = 0.802, *P* < .001) in Bangladesh. In contrast, literacy, activity, and poverty rate had a relatively weaker relationship with COVID-19 (Figure 1A). Only poverty was negatively associated with COVID-19 infection. Similar to bivariate statistics, the standardized coefficient of multiple linear regression also showed that population density was the significant predictor of COVID-19, which explains around 60% of the variation followed by the urbanization rate (20%) (Figure 1B). At the bivariate level, most of the predictors were significant, while at multiple analyses, only population density and urbanization were statistically significant and explained 80% of the model (all variables together explained 83%).

## Discussion

We aimed to assess the effect of population density on the spread of COVID-19 in Bangladesh. There was a strong positive correlation between population density and the number of COVID-19 infection in Bangladesh. A similar finding was observed in Algeria.^1^ Existing study also showed that connectivity matters more than density in the spread of the COVID-19 infection.^2^ In Bangladesh, the urban settlements have a higher risk of spreading diseases due to the enormous population density and connectivity. Group 4 districts (Dhaka and Chattogram) had the highest COVID-19 cases and were also the most populated cities of Bangladesh. A previous study also showed that metropolitan size mattered more than population density in the spread of COVID-19 in the United States.^3^

This study has some limitations. Some crucial predictors of COVID-19, including temperature, were not considered due to the unavailability of district-wise data. There are only 64 districts in Bangladesh, and it is a small sample. Sub-district-wise analysis may produce more stable and generalizable results.

Bangladesh has used nontherapeutic measures to reduce the numbers of COVID-19 confirmed cases in the absence of vaccine and treatment.^4-6^ However, due to higher population density, unplanned urbanization, and diverse socioeconomic conditions, these measures were not as effective as hoped for. The population density was the most crucial predictor of infection rates. For future planning of nontherapeutic measures to prevent COVID-19 and other related infectious diseases in Bangladesh, population density should be considered a risk factor.
